# Unilateral leukemic infiltration and acute angle closure as the first sign of B-cell acute lymphoblastic leukemia relapse

**DOI:** 10.3205/oc000105

**Published:** 2019-04-26

**Authors:** Diana Silva, Mafalda Mota, Andreia Bilé, Mário Ramalho, Sara Pinto, Graça Pires, Susana Teixeira, Isabel Prieto

**Affiliations:** 1 Hospital Prof. Dr. Fernando Fonseca E.P.E., Ophthalmology Department, Amadora-Sintra, Portugal; 2 Hospital Prof. Dr. Fernando Fonseca E.P.E., Pediatric Department, Amadora-Sintra, Portugal

**Keywords:** acute angle closure, B-cell acute lymphoblastic leukemia (ALL-B), ocular infiltration, relapse

## Abstract

**Objective:** Unilateral ocular leukemic infiltration with acute angle closure is an infrequent complication of B-cell acute lymphoblastic leukemia (ALL-B). We present a clinical case of leukemic ocular infiltration as the sole manifestation of ALL-B relapse.

**Methods:** Case description

**Results:** A 15-year-old female with a history of acute lymphoblastic leukemia in remission for 2 years and pulmonary tuberculosis treated in the past year presented with ocular redness and decreased visual acuity in the left eye (LE) with 5 days of evolution. Visual acuity was 20/20 in the right eye (RE) and absence of light perception in the left eye (LE). Biomicroscopy of LE showed a small hypopion, anterior chamber cells 4+, vitreous cells 3+, and a large white mass in the vitreous with associated vitreous hemorrhage in organization. In LE fundoscopy, the vitreous mass occupying most of the vitreous cavity and associated hemorrhage prevented retina visualization. B-scan ultrasound showed a multilobulated mass occupying virtually the entire vitreous cavity with associated choroidal detachment. Forty-eight hours later, she developed acute angle closure of LE with an IOP of 55 mmHg. A flow cytometric analysis of the anterior chamber and vitreous showed leukemic tumor cells. The microbiologic exam and PCR for *Mycobacterium tuberculosis* were negative. No other signs of relapse of the disease were identified after investigation by the oncology department. Rescue treatment of the underlying disease was started, with symptomatic improvement.

**Conclusion:** Leukemic ocular infiltration can be the only manifestation of ALL-B relapse.

## Introduction

Ophthalmic manifestations of leukemia have been described previously in literature and can occur in 9 to 90% of patients, mostly with retinal involvement [1], [2], [3]. Ophthalmic signs can be observed at the onset of disease or during follow-up [3], typically manifesting bilaterally and symmetrically [4]. However, it is uncommon that a relapsed disease presents solely with ocular manifestations [1], [4], [5]. We present a clinical case of unilateral leukemic ocular infiltration with choroidal infiltration and subsequent angle closure as the sole manifestation of ALL-B relapse.

## Case description

**Figure 1:**
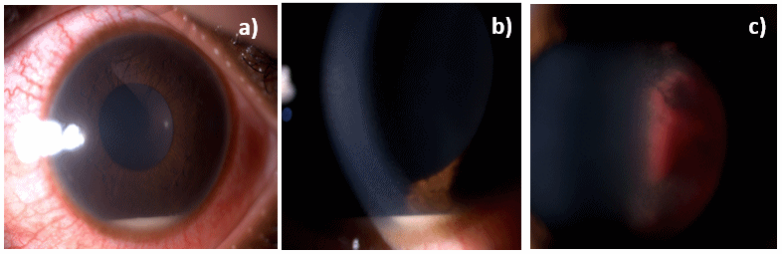
Biomicroscopy of the left eye showed a very small hypopyon, small, inferior, keratic precipitates, anterior chamber cells 4+, vitreous cells 3+, and a dense white mass was visible in the vitreous cavity along with organized vitreous hemorrhage.

**Figure 2:**
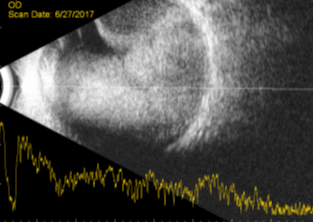
B-scan ultrasound of the left eye showing a multilobulated vitreous mass occupying practically the entire ocular globe with associated superior choroidal detachment

**Figure 3:**
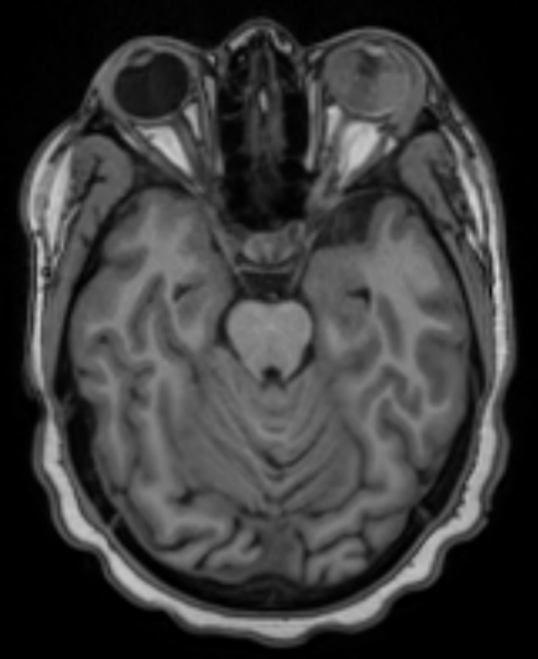
Orbital and cranial MRI revealed an intraocular mass with gadolinium enhancement and lacrimal gland enlargement. No signs of CNS metastasis were found.

We present the case of a 15-year old girl with a history of B-cell acute lymphoblastic leukemia (t(1:19)) in clinical remission for two years. She was under maintenance chemotherapy with dexamethasone, mercaptopurine, and melphalan. In the past year, she was also suffering from pulmonary tuberculosis having completed 9 months of therapy. She presented in our ophthalmology emergency room with acute visual loss of the left eye (LE) and ocular redness for 5 days. Best corrected visual acuity in her RE was 20/20 and absence of light perception in her left eye (LE). Intraocular pressure upon presentation was 12 mmHg in RE and 22 mmHg in LE. In LE biomicroscopy, she presented with a very small hypopyon, small, inferior, keratic precipitates, anterior chamber cells 4+, vitreous cells 3+, and a dense white mass was visible in the vitreous cavity along with organized vitreous hemorrhage (Figure 1 (Fig. 1)). The fundoscopy examination was made difficult by the dense vitritis. It was possible to perceive the presence of a white vitreous mass occupying most of the vitreous cavity and vitreous hemorrhage. Biomicroscopy and fundoscopy were normal in LE. B-scan ultrasound showed a multilobulated vitreous mass occupying practically the entire ocular globe with associated superior choroidal detachment (Figure 2 (Fig. 2)). An orbital and cranial MRI revealed an intraocular mass with gadolinium enhancement and lacrimal gland enlargement (Figure 3 (Fig. 3)). 

The patient was medicated with an association of Timolol 1% and Dorzolomide 1% bid as well as topical Dexamethasone 1% qid. Two days later, she suffered clinical worsening with severe ocular pain, headache, and nausea. IOP was now 55 mmHg in LE and the biomicroscopy of LE showed a shallow anterior chamber. Acute angle closure was diagnosed, and we added topical Brimonidine 0.1% bid and Acetazolamide 500 mg qid to her therapeutic scheme. A flow cytometry analysis of the aqueous humor and vitreous revealed ALL-B cells sharing the immunophenotype with previously diagnosed leukemic cells (CD34–/CD10+/CD19+/CD20+ weak/CD38+). Microbiologic analysis was negative for bacteria and fungus as well as the PCR study for *Mycobacterium tuberculosis*. Ocular relapse of B-cell ALL was diagnosed. The patient was re-staged but there were no signs of relapse in the bone marrow, the central nervous system or any other systemic location. Radiotherapy (30Gy) and rescue chemotherapy (INTREALL HR) were started with symptomatic improvement and better IOP control even though the patient was still under topical hypotensive drugs. Final MAVC was absence of light perception and 8 months after presentation, the patient remained without any other signs of systemic relapse.

## Discussion

We present a case of unilateral leukemic infiltration as the sole manifestation of relapsed ALL-B. Though, infrequent, unilateral eye involvement as the first sign of leukemic relapse has been described previously in the literature [2], [5], [6], [7]. Our patient presented with an intraocular mass occupying the entire vitreous cavity with associated vitreous hemorrhage and choroidal detachment. Forty-eight hours after presenting in our emergency room, the patient evolved to acute angle closure with an IOP of 55 mmHg, secondary to the pushing effect on the iris of the very extensive vitreous mass and associated choroidal detachment. Secondary acute angle closure as a manifestation of leukemic infiltration is very rare, with only two other cases previously described in the literature [6], [8]. In one of the cases, the patient already had an established diagnosis of AML at the time of visual symptoms and presented with bilateral disease [8]. In the other case, the patient was in remission for several years and presented with unilateral disease [6], making the diagnosis more challenging as in our case. 

Our patient’s case also had the particularity of a challenging differential diagnosis given that our patient was under maintenance chemotherapy and had history of a tuberculosis infection in the previous year. Therefore, alternative diagnostic hypothesis such as endogenous endophthalmitis and ocular tuberculosis had to be excluded as well, especially given that the patient was clinically in remission. The microbiologic exam and PCR for *Mycobacterium tuberculosis* were negative and the finding of leukemic cells in the vitreous and anterior chamber was diagnostic. 

Treatment of leukemic ocular infiltration comprises systemic chemotherapy, but if this is not an option or if the ocular disease is not responsive, ocular radiation may be combined [2], [6], typically with a total dose of 20 Gy [9]. Usually, it responds very well to systemic treatment, preventing the need for enucleation or evisceration [2], [5], [6], [7]. In our case, radiation therapy and chemotherapy improved the patient’s symptoms and decreased the intraocular mass. Initial visual acuity of absence of light perception at presentation suggests prolonged ocular involvement prior to the diagnosis hindering any possibility of visual recovery.

Findings of ocular leukemic relapse should prompt a new systemic work-up by the oncology team. Even though our patient still has not developed any signs of systemic leukemic relapse, in some cases where ocular findings were the only sign of leukemic relapse, bone marrow or CNS relapse ensued in the following months [5].

This case report highlights ocular leukemic infiltration as the first sign of leukemic relapse. A high suspicion index should be kept in these patients, even if in clinical remission for several years. Secondary acute angle closure is a less frequent presentation that can happen especially in cases with extensive intraocular infiltration.

## Notes

### Competing interests

The authors declare that they have no competing interests.
